# Community and family relationships across the transition to medical school: links to student adjustment

**DOI:** 10.3389/fpsyg.2024.1330455

**Published:** 2024-09-06

**Authors:** Brenda Rincon, Diamond Y. Bravo, Elisha Arnold, Alexis Meza, Daisy Camacho-Thompson, Chelsea D. Williams

**Affiliations:** ^1^Department of Psychology, University of California, Riverside, Riverside, CA, United States; ^2^Department of Psychology, California State University, Los Angeles, Los Angeles, CA, United States; ^3^Department of Psychology, Virginia Commonwealth University, Richmond, VA, United States

**Keywords:** medical students, field belonging, grade expectations, community support changes, family support changes

## Abstract

**Introduction:**

Supporting students during the transition to medical school is crucial for their academic adjustment. However, there has been limited research on the protective role of community and family support during this transition, despite evidence of the benefits of supportive relationships in higher education. Guided by self-determination theory, the current cross-sectional study explored how changes in family and community relationships impact Cuban medical students' sense of belonging in their field and their grade expectations.

**Methods:**

A total of 881 medical students (*M*_Age_ = 21.51, *SD*= 2.23, range = 18–33; 54% female; 72.2% Cuban) participated in this study. Participants included students across 6 years of medical school (1^st^ year = 14.8%, 2^nd^ year = 10%, 3^rd^ year = 24.1%, 4^th^ year = 19.3%, 5^th^ year = 11.8%, 6^th^ year = 20%).

**Results:**

Controlling for key demographics (e.g., student aid experience, family legacy, nationality, year in medical school, prior academic performance, gender, and offspring), our findings revealed that improvements in community relationships—rather than family relationships—were associated with higher levels of field belonging. Additionally, more extensive experience as a student aid and a greater number of family members with a medical background were linked to higher levels of field belonging and higher grade expectations. Notably, higher prior academic performance was associated with increased field belonging but did not affect grade expectations. Conversely, identifying as female was related to both higher field belonging and higher grade expectations.

**Discussion:**

Our study highlights the importance of supportive community relationships for medical students' academic adjustment. We discuss the potential benefits of enhancing community relationships within medical school training programs.

## 1 Introduction

Navigating the demanding medical school environment can be particularly challenging for students who are underrepresented in the field (e.g., low-income, first-generation, and students of color). This experience often leads to increased mental health distress and emotional challenges, adversely impacting academic adjustment (Rotenstein et al., 2016). While much research has explored barriers faced by medical students, the role of supportive relationships with family and community has received less attention. Despite evidence highlighting the benefits of social support in higher education, such as its protective effect against burnout and depression among U.S. medical students (Thompson et al., 2016), there is limited information on how these support relationships evolve during the transition to medical school.

Guided by self-determination theory (Deci and Ryan, 1985), the current study addresses this gap in the literature by examining changes in community and family relationships during the transition to medical school and their impact on students' academic adjustment (i.e., grade expectations, field belonging). Understanding how these social relationships evolve can help programs tailor their curricula to better support students, leading to more targeted and effective assistance throughout medical school.

### 1.1 Social support and academic adjustment during medical school

Self-determination theory (Deci and Ryan, 1985) explains the protective nature of social support by emphasizing three basic psychological needs: relatedness, autonomy, and competence. These needs are crucial for fostering motivation and persistence among students (Ryan and Deci, 2017). Social support satisfies the need for relatedness by providing emotional connections and a sense of belonging. It also enhances autonomy by promoting independence and self-direction, and supports competence by offering resources, feedback, and reinforcement that bolster individuals' confidence and effectiveness. Meeting these psychological needs is essential for persistence, psychological health, and academic adjustment (Ryan and Deci, 2017). In medical students, social support is particularly important due to the high levels of stress, depression, and coping challenges they often face during their training (Hill et al., 2018). Further exploration in this area can help improve support strategies for maintaining student wellbeing and academic success throughout medical school.

Transitioning to medical school involves adapting to rigorous academic demands, financial pressures, and balancing study, clinical rotations, and personal life, all within a high-stress environment that can lead to burnout, mental health issues (Hill et al., 2018), and imposter syndrome (i.e., self-doubt; Alsaleem et al., 2021). The shift from theoretical learning to hands-on patient care during clinical rotations, combined with potential social isolation and neglect of personal wellbeing, further complicates this transition (Hill et al., 2018; Bergmann et al., 2019). Given the stress and uncertainty of this phase, strong social support may buffer against these pressures and associated risks (e.g., dropout). While research on social support during this transition is limited, studies with college students indicate that support from community and family is crucial. Such support provides emotional, financial, and instrumental aid, enhancing academic experiences, particularly for low-income and marginalized populations (Roksa and Kinsley, 2019).

Community relationships, including support from peers, mentors, and educational institutions, are crucial for students' sense of belonging and access to academic and emotional resources (Nuñez, 2009; Maunder, 2018; Parnes et al., 2020). Peer support can be particularly valuable for medical students, as peers facing similar challenges can provide empathy, perspective, and practical advice (IsHak et al., 2013). Support from family relationships are also essential, offering emotional sustenance, encouragement, and a respite from academic pressures (Nichols and Islas, 2016; LeBouef and Dworkin, 2021). Stability in high levels of support or improvements in family and community relationships during the transition to medical school may therefore enhance students' academic adjustment and sense of belonging because such support directly addresses their psychological needs for relatedness, autonomy, and competence. This comprehensive support system can alleviate stress, promote resilience, and facilitate a more adaptive and fulfilling academic experience.

#### 1.1.1 Academic adjustment

Academic adjustment during the transition to medical school encompasses various factors, including a student's sense of belonging and their grade expectations. *Field belongingness*—the feeling of being accepted and included within the medical community—and *grade expectations*—anticipated academic performance—are crucial for successful academic adjustment. The significance of these factors is well-established in higher education. For example, a strong sense of field belonging among surgical residents has been linked to reduced thoughts of leaving their residency, suggesting that belongingness may protect against attrition (Salles et al., 2019). Similarly, academic expectations have been shown to correlate with academic achievement, as they influence student motivation and performance (Beal and Crockett, 2010; Khattab, 2015; Khattab and Modood, 2018). Despite the recognized importance of belongingness and grade expectations for academic success, there is limited research on how supportive relationships with family and community impact these factors during the transition to medical school. Understanding how changes in these relationships affect students' sense of belonging and grade expectations is crucial, as shifts in support structures can significantly influence academic adjustment and performance. The following section will address the role of supportive relationships and how changes in family and community support during the transition to medical school may shape students' academic outcomes and sense of belonging.

### 1.2 Changes in supportive relationships during the transition to medical school

Understanding how community and family relationships evolve as students transition to medical school is essential. Previous research has established connections between social support and increased retention, promotion, and satisfaction in medical training (Farkas et al., 2019). As students navigate the demanding medical school journey, strong community and family support can be crucial in helping them thrive by providing emotional support and guidance (Apugo, 2017; Covarrubias et al., 2019). In the following section, we discuss the dynamics of social support from family and community during this transition. To date, research has not thoroughly investigated how these relationships change throughout the transition to medical school. By exploring these changes, we can gain valuable insights into optimizing support systems and enhancing medical students' academic adjustment.

#### 1.2.1 Changes in community relationships

Students' perceptions of their supportive community in medical education extend beyond the traditional educational environment, which typically includes teachers, school administrators, and staff. In community-based medical programs, especially in contexts like Cuba, where collaboration between medical students and patient communities is emphasized, the concept of “community” can encompass a diverse array of individuals. This broader definition includes patients, healthcare professionals, local organizations, family members, and the broader society, all connected through shared values, interests, and norms (LeBan et al., 2021). In this study, community relationships are considered broadly to capture these various dimensions.

While research on the impact of community support on medical students' adjustment is limited, and often focuses on benefits to communities from medical practitioners, emerging studies highlight the importance of community relationships for students. For instance, studies have shown that lack of community involvement and misalignment between personal career goals and community values can negatively impact premedical biology students' sense of belonging (Knekta and McCartney, 2021). Additionally, research with undergraduate college students has found that positive community interactions are linked to a higher sense of belonging, increased motivation, and a greater desire to engage in community activities (Booker, 2016). These findings underscore the potential role of supportive community relationships in enhancing medical students' academic experiences.

The current study builds on this body of research by examining how changes in community relationships are linked with the adjustment of Cuban medical students. Previous work has indicated that medical students' relationships with their communities evolve through various programs. For example, Cuban medical students involved in rural or community-based medical programs often report increased levels of community support and positive changes in their perceptions of the local population (Crump et al., 2019). Additionally, experiences in rural or underserved areas have been shown to improve medical students' attitudes toward these communities and their willingness to work in such settings (Kane et al., 2013). As students undergo the transition into medical school in Cuba, these evolving community relationships, along with potential changes in family dynamics, may significantly influence their academic adjustment and overall experience.

#### 1.2.2 Changes in family relationships

Family support is fundamental to medical students' academic adjustment, playing a key role in their emotional and practical wellbeing during the demanding transition to medical school (LeBouef and Dworkin, 2021). The transition often involves relocating away from home, which can alter the nature and frequency of family interactions. Students may become dependent on virtual communication to stay connected with their families, potentially impacting the quality of emotional support they receive (Guo et al., 2014). This shift can create a sense of distance, both physically and emotionally, which may affect students' sense of belonging and academic expectations.

Supportive family relationships have been shown to foster academic persistence, engagement, and psychological wellbeing among students (Nichols and Islas, 2016). For instance, first-generation college students often rely on family support to navigate academic challenges and maintain motivation (Azmitia et al., 2018). However, as students transition into medical school, changes in family dynamics—such as reduced face-to-face interactions or shifts in family roles—can significantly impact their academic experience and emotional adjustment (Lowe and Dotterer, 2018).

Despite the established importance of family support, it is crucial to explore how changes in family relationships influence academic outcomes compared to community support. While research has highlighted the role of community support in educational settings, there is limited understanding of how family support evolves during the transition to medical school and its relative impact. Investigating whether changes in family support or community support have a greater influence on students' academic adjustment will help clarify which support systems are most effective during this critical period.

### 1.3 Current study

**Figure 1:**
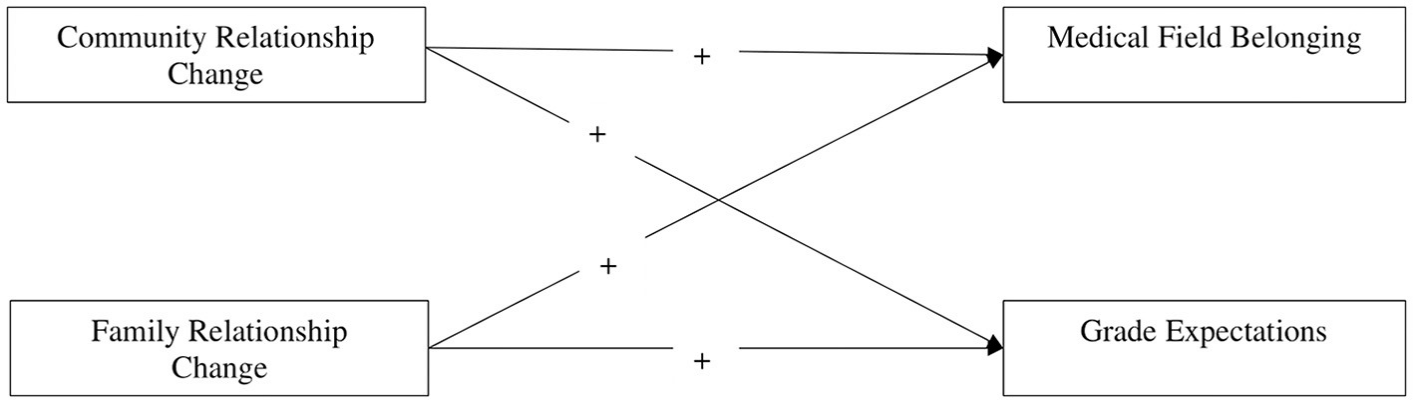
Hypothesized study associations. The hypothesized model includes student aid frequency, family legacy, nationality, year in medical school, previous academic performance, gender and whether are not participants have offspring as controls.

This study examines how changes in community and family support impact academic adjustment, specifically focusing on students' sense of belonging and grade expectations, among medical students in Cuba. We hypothesize that improvements in both community and family support will positively correlate with enhanced academic adjustment outcomes. The hypothesized model is illustrated in Figure 1.

To ensure a comprehensive analysis, we controlled for several factors: students' experience with medical-related work, family legacy, year of medical school, prior academic performance, parental status, nationality, and gender. We controlled for experience in medical-related work due to its potential influence on students' engagement, understanding of the medical field, and overall academic satisfaction (Coates et al., 2008; Bergmann et al., 2019). Family legacy was included to account for the potential impact of having family members in the medical field on students' sense of belonging and academic motivation (Baldwin et al., 2020). The year of medical school was included to account for variations in academic performance and challenges encountered at different stages of medical training (Pinyopornpanish et al., 2004). Prior academic performance was included as it is a strong predictor of future academic success (Ferguson et al., 2002). Parental status was considered due to its impact on balancing academic and familial responsibilities, which can influence academic workload management (Hill et al., 2018). Nationality was controlled because native-born students often report higher levels of sense of belonging compared to immigrant peers (Green, 2015; World Health Organization, 2018). Finally, gender was included in the analysis as it can impact students' sense of belonging, academic performance, and social support experiences, with differing effects observed in various contexts (Pololi et al., 2013).

## 2 Methods

### 2.1 Participants

The present study utilizes data from the survey component of the Cuban Medical Student Motivation Project (CMSMP). The CMSMP study employed an asset-based, mixed-method, grounded approach to investigate the cultural mechanisms underlying the experiences of medical students in Cuba. The study included 881 students (M_Age_ = 21.51, *SD* = 2.23; 54% female, 44% non-white) attending medical school in Cuba during the 2019–2020 academic school year. Data were collected across 6 years of enrollment (M_year_ = 3.6, *SD* = 1.67), and most participants identified as Cuban (72.2%, 636/881). The study did not measure race, as Cuban collaborators noted that skin color more accurately represented participants' conceptualizations of ethnic and racial identity experiences than race within the Cuban context. Skin color descriptives can be found in Table 1.

**Table 1:** Descriptive statistics for study and demographic variables among Cuban medical school students.

**Variable**	***N***	***M***	***SD***	**Range**	***n***	**%**
Field belonging	863	4.46	0.69	1–5		
Very much disagree					6	0.7
Disagree					7	0.8
Neither agree nor disagree					40	4.6
Agree					340	39.4
Very much agree					470	54.5
Grade expectations	781	2.59	0.6	1–3		
No					47	6
Same as previous year					229	29.3
Yes					505	64.7
Community relationship change	864	2.34	0.57	1–3		
Worsened					41	4.7
Stayed the same					490	56.7
Improved					333	38.5
Family relationship change	864	2.3	0.54	1–3		
Worsened					36	4.2
Stayed the same					536	62
Improved					292	33.8
Student aid work frequency	824	2.21	1.46	1–5		
Never					462	56.1
Yearly					36	4.4
Monthly					40	4.9
Weekly					259	31.4
Daily					27	3.3
Family legacy	864	0.88	0.87	0–3		
None (0)					333	38.5
Few (1–2)					357	41.3
Some (3–4)					122	14.1
Many (5 or more)					52	6
Nationality	772	–	–	–		
Cuban					636	82.4
Non-Cuban					136	17.6
Year in medical school	861	3.63	1.66	1–6		
1st year					127	14.8
2nd year					86	10
3rd year					208	24.2
4th year					166	19.3
5th year					102	11.7
6th year					172	20
Prior academic performance	791	3.32	0.57	1–4		
Very low					9	1.1
Low					17	2.1
Average					479	60.6
High					286	36.2
Gender	838	–	–	–		
Female					472	56.3
Male					366	43.7
Offspring	724	–	–	–		
No					684	94.5
Yes					40	5.5
Age	807	21.51	2.23	18–33		
18					33	4.1
19					114	14.1
20					151	18.7
21					143	17.7
22					130	16.1
23					101	12.5
24					63	7.8
25					45	5.6
26					8	1
27					6	0.7
28					1	0.1
29					6	0.7
30					3	0.4
31					0	0
32					2	0.2
33					1	0.1
Skin color	816	–	–	–		
Black					198	24.3
Mestiza					192	23.5
White					426	52.3

N denotes the number of participants that answered each question. Percentages are based on the number of participants that responded to the question. The total sample size for the study is N = 881.

### 2.2 Procedure

CMSMP was approved by a Cuban university's institutional review board. The sample size was determined to ensure sufficient statistical power for the analysis. With nine predictors and two outcome variables, we aimed for a minimum of 10–15 participants per predictor, as suggested by Cohen (1992). To achieve robust and reliable results, we targeted a sample size of at least 135 participants. Participants eligible for this study were required to meet three primary inclusion criteria. Participants were required to be currently enrolled in one of the five selected medical schools in Cuba, be at least 18 years old, and provide informed consent to participate in the study. The survey was distributed to all students enrolled at these five medical schools. We achieved a 100% response rate, as all contacted participants agreed to participate. The study utilized a comprehensive sampling approach by including students from five local medical schools, ensuring a representative sample of the medical student population in Cuba. Students completed the paper survey during their first-period class without a designated time limit. The survey spanned three pages and required ~20 min to complete. To maintain participant anonymity, no identifying information was collected following standard Cuban confidentiality protocols. Consistent with standard practices in Cuba, participants were not provided with any incentives for their involvement in the study. Four participants were excluded as they were under the age of 18. Open-ended questions were translated from Spanish to English and coded by a team of researchers.

### 2.3 Study variables

*Field belonging. Sense of medical field belonging* was measured using a single self-reported item (i.e., “I feel like I belong in the field of medicine; Siento que pertenezco a la Carrera de medicina”). Participants used a 5-point Likert Scale ranging from (1) *Very much disagree* to (5) *Very much agree* to indicate their perceived sense of belonging to the field of medicine. Higher scores indicate more sense of belonging.

*Grade expectations* were measured using a single item. Participants were asked about their grade expectations for the current year (i.e., “Do you think you will get higher grades this year; Considera que este año obtendra notas mejores?”). Participants responded using a 3-point Likert Scale including (1) *No*, (2) *Same as previous year* and (3) *Yes*.

*Community relationship change* was measured using a single-item indicator. Participants were asked about changes in community relationships since starting medical school (i.e., “Has your relationship with your community changed since starting medical school; Desde que comenzo la Universidad, como considera la relacion con su comunidad?”). Participants indicated relationship change regarding their community using a 3-point Likert Scale ranging from (1) *Worsened*, (2) *Stayed the same*, to (3) *Improved*. Betterment of community relations is indicated by higher scores.

*Family relationship change* was assessed using a single indicator. The item asked about changes in family relationships since starting medical school (i.e., “Has the relationship with your family changed since starting medical school; Desde que comenzo la Universidad, como considera la relacion con su familia?”). Changes to the family relationship were indicated using a 3-point Likert Scale of (1) *Worsened*, (2) *Stayed the same*, and (3) *Improved*. Strengthened family relationships are indicated by higher scores.

### 2.4 Study control variables

Study control variables include student aid work experience frequency, family legacy, nationality, year in medical school, prior academic performance, gender, and whether participants had offspring. To assess the frequency with which students worked within a medical specialty as a student aid, students were asked, “How often do you work as a student aid?” Participants responded using a five-item scale ranging from (1) never to (5) daily, with higher scores indicating working as a student aid more frequently. A single-item (e.g., “How many family members work in the field of medicine?”) was used to assess family legacy. Responses included 0 = None (0), 1 = Few (1-2), 2 = Some (3-4), and 3 = Many (5 or more), with higher scores indicating more family members working in the field of medicine. Additionally, the study controlled for *nationality* (0 = Cuban, 1 = non-Cuban) and *year in medical school* (1 = 1st year to 6 = 6th year). Academic performance from the previous year was self-reported by participants using a single item (e.g., “How would you rate your overall academic performance from the previous school year?”). Responses were coded as “1 = Very low,” “2 = Low,” “3 = Average,” and “4 = High.” Lastly, the study controlled for *gender* (0 = female, 1 = male) and whether participants had *offspring* (0 = no, 1 = yes).

### 2.5 Analytic strategy

Path analysis within a structural equation modeling framework was conducted with M*plus* version 8.8 (Muthén and Muthén, 2022). The cross sectional model examined links between changes in community relationships and changes in family relationships and medical students' adjustment (i.e., field belonging, grade expectations for the upcoming year). Moreover, measures of frequency working as a student aid, family legacy in medicine, year in medical school, prior academic performance, gender, nationality, and the number of offspring were included as controls. Common method bias was assessed using Harmon's single-factor test in SPSS version 29. The single-factor analysis did not exceed 50%, and therefore the model does not demonstrate common method bias. Additionally, all exogenous variables were allowed to covary. Full information maximum likelihood (FIML) was used to account for missing data (Enders, 2010). To estimate the model parameters, we employed maximum likelihood parameter estimation (ML) and a bootstrap method with *N* = 1,000 resamples. The approach allows us to approximate the sampling distribution of the statistic and derive confidence intervals around the estimate. The developed model is a fully saturated model, and therefore, model fit indices are *X*^2^ = 0, *df* = 0, *p* = 0, and CFI = 1.00.

## 3 Results

### 3.1 Descriptive results

The current study examined the link between changes in social support—specifically, changes in family and community relationships—and medical students' academic adjustment, measured as field belonging and grade expectations. Out of 881 participants, the majority reported no change (i.e., “stayed the same”) in their relationships with family (61%) and community members (56%). Among those who did experience relationship changes, the majority indicated improvements for both community relationship change (38.5%) and family relationship change (33.8%). Additionally, over half of the participants did not work as student aids (58%); however, those who did were typically employed on a weekly basis (72%). Most participants had 1–2 family members working in the medical field (41%), whereas 38% reported having no family members in medicine. Detailed descriptive statistics, including age, and skin color, are provided in Table 1.

### 3.2 Hypothesized associations

Our hypotheses regarding the impact of changes in community and family support on academic adjustment were partially supported. At the bivariate level, improvements in both community and family relationships were positively associated with a greater sense of belonging to the field of medicine. This suggests that students who experienced positive changes in these relationships were more likely to report a stronger connection to their field (see Table 2). Moreover, while positive changes in family relationships correlated with higher grade expectations, changes in community relationships did not show a significant correlation with this outcome.

**Table 2:** Correlations for study variables among Cuban medical students (*N* = 881).

**Variables**	**1**	**2**	**3**	**4**	**5**	**6**	**7**	**8**	**9**	**10**	**11**
1. Field belonging	—										
2. Grade expectations	0.22^***^	—									
3. Community relationship change	0.12^***^	0.02	—								
4. Family relationship change	0.11^**^	0.08^*^	0.33^***^	—							
5. Student aid work frequency	0.20^***^	0.14^***^	0.11^**^	0.11^**^	—						
6. Family legacy	0.09^*^	0.08^*^	−0.03	0.02	0.03	—					
7. Nationality^a^	−0.06	−0.04	−0.23^***^	−0.11^**^	−0.18^***^	−0.03	—				
8. Year in medical school	0.02	−0.02	0.14^***^	0.08^*^	0.20^***^	−0.02	0.05	—			
9. Prior academic performance	0.20^***^	0.05	0.10^**^	0.03	0.26^***^	0.07	−0.03	0.15^**^	—		
10. Gender^b^	−0.11^**^	−0.08^*^	−0.01	−0.06	−0.03	0.01	−0.03	−0.04	−0.11^**^	—	
11. Offspring^c^	−0.01	−0.08	0.01	0.03	0.08^*^	0.01	−0.02	0.05	−0.03	0.08^*^	—

^a^0 = Cuban, 1 = non-Cuban.

^b^0 = female, 1 = male.

^c^0 = no, 1 = yes.

^*^p < 0.05.

^**^p < 0.01.

^***^p < 0.001.

**Figure 2:**
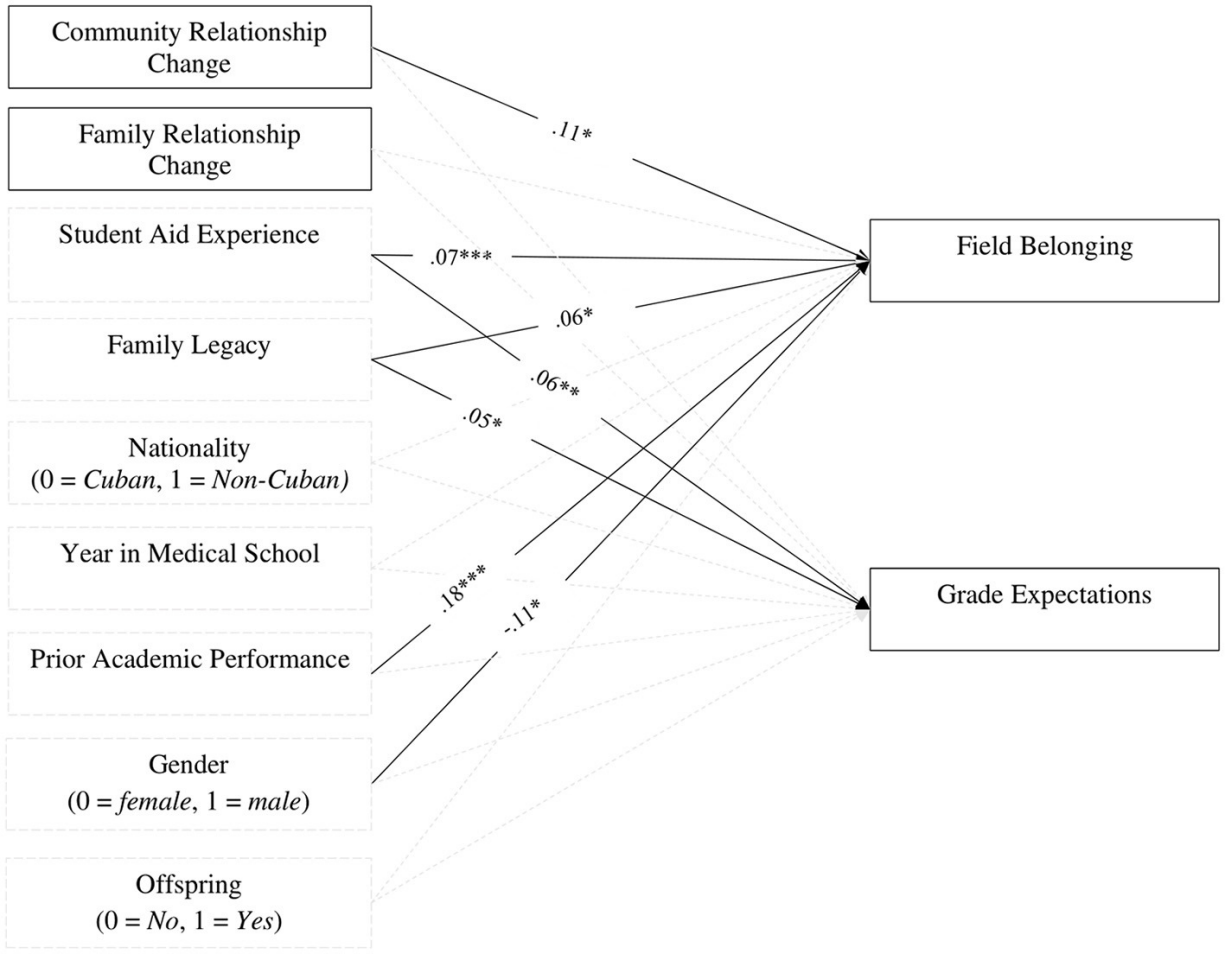
Crosssectional associations between changes in social support and academic adjustment. Model fit indices not available for fully saturated model. Unstandardized coefficients displayed. Family legacy was coded as 0 = None (0), 1 = Few (1–2), = Some (3–4), and 3 = Many (5 or more). Year in medical school included those in their 1st year (14.8%), 2nd year (10%), 3rd year (24.1%), 4th year (19.3%), 5th year (11.8%), and 6th year (20%), Grade expectations was coded as 1 = No, 2 = Same as previous year, and 3 = Yes. Control variables are displayed in gray dashed boxes. Gray dashed arrows denote non-significant path coefficients. ***p < 0.001, **p < 0.01, and *p < 0.5.

Next, regression analyses showed that improved community relationships were positively associated with a higher sense of belonging to the field of medicine (see Figure 2 and Table 3). However, changes in family relationships did not significantly predict either field belonging or grade expectations. The variance explained by the predictors for field belonging was *R*^2^ = 0.092, indicating that 9.2% of the variance was accounted for by the included variables, such as community relationship change, student aid experience, family legacy, prior academic performance, and gender. For grade expectations, the explained variance was *R*^2^ = 0.041, meaning that 4.1% of the variance was explained by the same set of predictors.

**Table 3:** Regression analysis with bootstrapped confidence intervals predicting field belonging and grade expectations.

	**Field belonging**						**Grade expectations**					
**Predictor variable**	**B**	**SE**	**95% CI lower**	**95% CI upper**	β	***p***	**B**	**SE**	**95% CI lower**	**95% CI upper**	β	***p***
Intercept	3.383	0.209	2.975	3.792	-	0	2.355	0.184	1.994	2.715	-	0
Community relationship change	0.105	0.044	0.017	0.155	0.086	0.015	0	0.04	−0.073	0.073	0	1
Family relationship change	0.063	0.049	−0.027	0.125	0.049	0.203	0.071	0.041	−0.009	0.136	0.063	0.088
Student aid experience	0.071	0.017	0.081	0.218	0.149	< 0.001	0.056	0.017	0.056	0.212	0.134	0.001
Family legacy	0.057	0.026	0.008	0.135	0.072	0.027	0.052	0.025	0.005	0.144	0.075	0.036
Nationality	−0.001	0.074	−0.081	0.08	−0.001	0.988	−0.010	0.069	−0.092	0.079	−0.006	0.882
Year in medical school	−0.020	0.014	−0.114	0.015	−0.049	0.132	−0.014	0.014	−0.116	0.039	−0.038	0.33
Previous academic performance	0.184	0.053	0.066	0.242	0.154	0.001	0	0.046	−0.087	0.087	0	0.994
Gender	−0.114	0.049	−0.150	−0.015	−0.082	0.017	−0.091	0.046	−0.149	0	−0.075	0.05
Offspring	−0.044	0.104	−0.081	0.052	−0.015	0.669	−0.197	0.12	−0.164	0.015	−0.075	0.102

B, unstandardized coefficient; SE, standard error; CI, confidence interval; β, standardized coefficient; p, p-value.

Confidence intervals were calculated using bootstrapping with 1,000 resamples. N = 881.

Finally, several control variables were significantly associated with the study outcomes. Increased student aid experience, having a family legacy in medicine, and identifying as female were linked to higher grade expectations (see Figure 2 and Table 3). Additionally, greater student aid experience, higher family legacy, and prior academic performance were associated with a stronger sense of belonging to the field of medicine (see Table 3).

### 3.3 *Post-hoc* analyses

To better understand the impact of relationship changes on academic outcomes, we conducted *post-hoc* ANOVAs following our initial analyses. These tests aimed to explore how different types of relationship changes—worsened, unchanged, or improved—in both family and community contexts were associated with variations in field belonging and grade expectations, and student contextual experiences (see Table 4).

**Table 4:** Descriptive statistics and ANOVA results for community and family relationship changes.

		**Community relationship change**								**Family relationship change**							
**Variable**	***N***	**Worsened (*****n*** = **41)**		**Stayed the same (*****n*** = **490)**		**Improved (*****n*** = **333)**				**Worsened (*****n*** = **36)**		**Stayed the same (*****n*** = **536)**		**Improved (*****n*** = **292)**			
		**M**	**SD**	**M**	**SD**	**M**	**SD**	***F***	***p*** **-value**	**M**	**SD**	**M**	**SD**	**M**	**SD**	***F***	***p*** **-value**
Field belonging	863	4.43	0.71	4.38^A^	0.73	4.58^A^	0.62	8.17	< 0.001	4.23^A^	0.91	4.43^B^	0.65	4.55^A, B^	0.72	4.9	0.008
Grade expectations	781	2.62	0.54	2.56	0.6	2.61	0.61	0.52	0.6	2.62	0.6	2.54^A^	0.61	2.66^A^	0.59	3.58	0.03
Community relationship change	864	-	-	-	-	-	-	-	-	1.7^A, B^	0.59	2.26^A, C^	0.52	2.56^B, C^	0.55	53.66	< 0.001
Family relationship change	864	1.9^A, B^	0.71	2.19^A, C^	0.48	2.5^B, C^	0.51	50.22	< 0.001	-	-	-	-	-	-	-	-
Student aid experience	824	2.14	1.4	2.07^A^	1.41	2.43^A^	1.5	5.87	0.003	2.26	1.46	2.08^A^	1.41	2.47^A^	1.51	6.3	0.002
Family legacy	864	0.93	0.82	0.89	0.87	0.84	0.87	0.45	0.64	0.78	0.8	0.87	0.87	0.89	0.86	0.31	0.74
Nationality	772	0.61^A, B^	0.49	0.19^A, C^	0.39	0.1^B, C^	0.3	32.31	< 0.001	0.48^A, B^	0.51	0.18^A^	0.38	0.14^B^	0.35	10.7	< 0.001
Year in medical school	861	4.07^A^	1.85	3.33^A, B^	1.66	4.02^B^	1.54	19.14	< 0.001	3.58	1.75	3.54^A^	1.64	3.83^A^	1.66	3	0.05
Previous academic performance	791	3.33	0.53	3.25^A^	0.59	3.39^A^	0.56	5.45	0.004	3.21	0.6	3.32	0.57	3.33	0.58	0.59	0.55
Gender	838	0.43	0.5	0.43	0.5	0.43	0.5	0.01	0.99	0.51	0.51	0.45	0.5	0.4	0.49	1.46	0.23
Offspring	724	0.08	0.28	0.05	0.21	0.06	0.24	0.65	0.53	0	0	0.06	0.24	0.06	0.24	0.93	0.39

N, sample size; M, mean; SD, standard deviation; F, F-ratio; p, p-value.

ANOVA and Tukey HSD *post-hoc* tests were conducted to assess group differences by community relationship change and family relationship change. Means with the same superscript indicate significant Tukey HSD results at p < 0.05.

To identify specific group differences, Tukey's Honest Significant Difference (HSD) *post-hoc* tests were conducted. Significant differences emerged across various dimensions. Regarding field belonging, students who reported improved community relationships exhibited higher levels of field belonging compared to those whose community relationships stayed the same. Conversely, students with worsened community relationships reported lower levels of field belonging compared to those with unchanged community relationships. In terms of family relationships, students with worsened community relationships experienced less positive changes in their family relationships compared to those whose community relationships stayed the same or improved. Similarly, students whose community relationships remained the same reported less favorable changes in their family relationships compared to those whose community relationships improved.

Next, regarding student aid experience, students who reported improvements in community relationships worked as student aids more frequently than those whose community relationships stayed the same. Nationality differences also emerged: students with worsened community relationships were more likely to identify as non-Cuban compared to those whose community relationships remained unchanged or improved. Conversely, students whose community relationships improved were more likely to identify as Cuban compared to those whose relationships stayed the same. There were also differences by year in medical school, with students whose community relationships remained unchanged being more likely to be in the earlier years of the program compared to those with worsened or improved relationships. Finally, students who reported improvements in their community relationships had higher prior academic performance compared to those whose community relationships stayed the same.

Changes in family relationships revealed several significant differences. Students who experienced worsened family relationships had lower levels of field belonging compared to their peers who reported improvements in these relationships. Similarly, those whose family relationships remained unchanged reported lower field belonging than those who noted improvements. In terms of grade expectations, students with improved family relationships had more positive expectations compared to those whose family relationships did not change. Significant differences also emerged in community relationship changes based on family relationship changes. Students with worsened family relationships reported less favorable changes in community relationships than those with unchanged or improved family relationships. Conversely, those who saw improvements in family relationships also reported more positive changes in community relationships compared to students whose family relationships stayed the same. Additionally, students with improved family relationships tended to work as student aids more frequently than those with stable family relationships. Finally, students who experienced worsened family relationships were more likely to be non-Cuban compared to those whose family relationships remained the same or improved.

## 4 Discussion

This study examined the impact of changes in community and family support on medical students' academic adjustment, specifically focusing on their belonging to the field of medicine and grade expectations. In light of ongoing efforts to improve medical student retention and success, it is imperative to consider comprehensive support strategies that extend beyond the university environment (Blalock et al., 2021). Grounded in self-determination theory (Deci and Ryan, 1985), this research offers a novel examination of how variations in social support—particularly changes in family and community relationships—affect academic outcomes for medical students in Cuba.

Our findings underscore the pivotal role of community support in influencing academic adjustment. Consistent with self-determination theory, improvements in community support were identified as a significant predictor of a stronger field belonging among medical students. In contrast, changes in family relationships did not exhibit a significant predictive relationship with academic outcomes. This finding highlights that, within the context of this study, community support emerged as a more critical factor for predicting students' field belonging and grade expectations, compared to changes in family relationships. Additionally, students with more extensive involvement in student aid roles reported a stronger connection to the medical field and higher grade expectations. This suggests that the depth of engagement in student aid activities—rather than the frequency of involvement—can positively influence students' academic outlook and sense of belonging. These findings have important implications for educational programs aiming to enhance support and retention rates among medical students. The evidence points to the necessity of emphasizing community support and providing substantive opportunities for student engagement as strategies to improve academic adjustment and success.

### 4.1 Linking changes to community and family support to students' academic adjustment

Our initial hypotheses posited that significant improvements in both community and family relationships upon entering medical school would enhance students' sense of affiliation with the medical field and elevate their grade expectations. While our findings partially supported these hypotheses, with enhanced community relationships and increased student aid experience predicting positive academic adjustment, improvements in family relationships did not have the anticipated link with these outcomes.

#### 4.1.1 Community support

Our findings align with prior research emphasizing the importance of community relationships in fostering a sense of belonging within academic settings (Booker, 2016; Knekta and McCartney, 2021). The study revealed that improvements in community relationships were positively associated with stronger belonging to the medical field. This reinforces the notion that a supportive community plays a crucial role in helping students integrate into their professional environment. The lack of a significant association between community relationships and grade expectations suggests that while community support is vital for fostering field belonging, it may not directly impact academic performance. This is consistent with previous findings that suggest a sense of belonging is more closely linked to retention and overall wellbeing rather than immediate academic outcomes (Tinto, 1993; Strayhorn, 2018).

Our results underscore the role of community networks in enhancing students' professional identity and integration, a key factor in their academic journey. This perspective is supported by self-determination theory, which posits that fulfilling the need for relatedness, as met through supportive community relationships, can significantly impact motivational outcomes (Deci and Ryan, 1985). The lack of direct impact on grade expectations could be attributed to the complex and multifaceted nature of academic performance, which is influenced by a broader range of factors beyond immediate community interactions.

#### 4.1.2 Family support

Our initial hypotheses expected that improvements in family relationships would significantly correlate with higher levels of medical students' field belonging and grade expectations. However, our findings did not fully support these predictions. Although there was a notable correlation between changes in family relationships and both outcome variables at the bivariate level, these correlations weakened when accounting for changes in community relationships. This suggests a complex interplay between familial and community relationships in shaping students' academic adjustment.

When considered independently, changes in family relationships did show some correlation with students' field belonging and grade expectations. However, once community relationships—encompassing interactions with various groups and networks beyond just the academic sphere—were factored in, the correlation between family relationship changes and the academic outcomes diminished. This indicates that relationships within broader community contexts may have a more significant association with students' field belonging and academic expectations.

The reduced correlation between family relationship changes and academic adjustment suggests that community relationships might have a more substantial impact in this context. Community relationships, which may include interactions with teachers, patients, local practitioners, and other social networks, may be more influential due to their direct and frequent engagement with students' daily experiences and identity formation. These findings underscore the need for further research to explore the nuanced ways in which different forms of social support relate to medical students' academic experiences and outcomes, highlighting the potentially central role of various community relationships.

### 4.2 Insights from *post-hoc* analyses: impact of relationship changes on academic adjustment

Our *post-hoc* analyses revealed that students with improved community relationships generally exhibited more positive academic adjustment. Specifically, those whose community relationships improved showed higher levels of field belonging and more favorable grade expectations compared to those whose relationships remained unchanged or worsened. In terms of family relationships, students who experienced improvements also had better academic adjustment, though these effects were less pronounced. These results suggest that positive changes in community relationships are particularly influential in enhancing students' field belonging and grade expectations.

#### 4.2.1 Community relationships and academic adjustment

Our findings demonstrate that improvements in community relationships are significantly associated with higher levels of field belonging. Students who reported enhanced community support felt a stronger connection to the medical field, which is consistent with previous research showing that social networks play a crucial role in fostering field belonging and academic engagement (Booker, 2016; Knekta and McCartney, 2021). Conversely, students with worsened community relationships experienced lower levels of field belonging and engaged less frequently in student aid roles. This suggests that deteriorated community support can undermine students' integration into their academic environment and their involvement in educational activities.

Of note, students whose community relationships remained unchanged were more likely to be in the earlier stages of their medical education and showed lower prior academic performance. This finding indicates that the impact of community support might become more significant as students advance in their training. Additionally, the data revealed that non-Cuban students were more likely to experience worsened community relationships compared to their Cuban peers. This could reflect cultural or contextual differences in the way community support impacts academic experiences, highlighting the need for culturally sensitive support strategies.

#### 4.2.2 Family relationships and academic outcomes

The study also underscores the importance of family support in academic adjustment. Improvements in family relationships were positively correlated with both field belonging and grade expectations. Students who experienced better family support felt more connected to their medical field and had more optimistic grade expectations, aligning with previous research emphasizing the role of familial support in academic success (Fuchs and Diamantopoulos, 2009). Conversely, students with worsened family relationships reported lower levels of field belonging and less favorable grade expectations.

Interestingly, our findings revealed that students with worsened family relationships reported the lowest scores for field belonging. This outcome highlights the significant role family support plays in a student's sense of integration and connection within their academic environment. The deterioration of family relationships may undermine the emotional and psychological support crucial for a positive educational experience, potentially leading to decreased motivation and a diminished field belonging. The impact of worsened family relationships on field belonging could be attributed to the essential role families often play in providing emotional support and stability. When family dynamics deteriorate, students might experience increased stress and anxiety, which can detract from their ability to engage fully with their academic community. This emotional strain may lead to lower levels of field belonging, as students may struggle to find a supportive network within their academic sphere when their familial support is compromised. Additionally, the lack of family support might impact students' ability to handle academic pressures effectively, further affecting their overall academic performance and field belonging. These findings underscore the importance of family relationships in shaping students' academic experiences and suggest that efforts to support medical students should consider not only the enhancement of community relationships but also strategies to address and mitigate challenges within family contexts. Future research should explore these dynamics further to understand how changes in family relationships specifically influence academic adjustment and identify potential interventions to support students facing such challenges.

Finally, students with improved family relationships also reported better community support, while those with worsened family relationships experienced less favorable changes in community interactions. This interdependence suggests that efforts to enhance family support may also positively influence community relationships, and vice versa. Thus, interventions that simultaneously address family and community support could be particularly effective.

### 4.3 Study limitations and future directions

This study offers important insights into the role of social support in medical students' academic adjustment but has several limitations that warrant consideration. First, the cross-sectional design of the study restricts our ability to infer causal relationships between changes in community and family relationships and academic adjustment outcomes. As data were collected at a single point in time, it is challenging to discern how these relationships evolve and impact academic adjustment over the course of medical education. Another notable limitation is the reliance on single-item measures, which may limit the depth and reliability of the data. Single-item measures, while practical, often lack the detailed insight provided by multi-item scales, potentially affecting the granularity and consistency of the findings (Fuchs and Diamantopoulos, 2009). This choice was influenced by cultural and logistical factors specific to the Cuban context, where single-item measures align with local practices and enhance the study's ecological validity and participant engagement (Diaz et al., 2015). Additionally, the study did not differentiate among various facets of social support, such as emotional, instrumental, and economic support, which could provide a more nuanced understanding of the changes in social relationships during the transition to medical school. A more detailed examination of these different types of support could illuminate how specific aspects of social support contribute to academic adjustment.

To address these limitations and build on the findings, future research should employ longitudinal designs to track changes over time and better identify causal relationships. Additionally, using multi-item scales and more detailed measures of specific types of social support would offer a deeper understanding of how different forms of support influence academic adjustment. Including more varied response options, such as Likert scales with multiple points, could capture a more nuanced representation of participants' experiences. Furthermore, future studies should explore potential barriers to maintaining or strengthening relationships during medical school, such as limitations in communication resources or time constraints. Understanding these barriers would provide a more comprehensive view of the challenges faced by medical students and enhance our understanding of the dynamics influencing academic adjustment.

### 4.4 Conclusion

Findings from the current study highlight the significant role of community support in the academic adjustment of medical students in Cuba, a country with one of the highest concentrations of medical students globally. Our findings reveal that improvements in community relationships are strongly associated with a heightened field belonging among medical students. This underscores the critical importance of robust community engagement and support systems in facilitating students' academic and professional development. Cuba's unique educational context, characterized by its high density of medical students and strong community-based training, provides valuable insights into how social support impacts academic adjustment. This research, focusing on an understudied population, offers a fresh perspective on the ways in which community connections contribute to students' field belonging and academic success.

Although changes in family relationships did not show a direct impact on academic adjustment outcomes in this study, the data indicate that community support plays a more pronounced role. This finding suggests that efforts to enhance students' integration into their communities can be particularly beneficial. Medical schools should therefore prioritize creating opportunities for students to engage meaningfully with the communities they serve, as these interactions are vital for fostering a strong sense of field belonging and improving academic outcomes. In light of the unique context of Cuba and the specific strengths of this study, it is recommended that future research continue to explore the role of community support in different educational settings. Additionally, further investigation into the impact of family support is needed to gain a comprehensive understanding of its potential effects on students' academic experiences. Such research will be essential for developing targeted strategies that support the diverse needs of medical students globally. Overall, this study provides important insights into the dynamics of community support and its impact on medical students' academic adjustment. The findings emphasize the need for continued exploration and application of these insights to enhance support systems and promote the success of medical students from diverse backgrounds.

## Data Availability

The raw data supporting the conclusions of this article will be made available by the authors, without undue reservation.
