# Correction: Community and family relationships across the transition to medical school: links to student adjustment

**DOI:** 10.3389/fpsyg.2025.1646170

**Published:** 2025-07-23

**Authors:** Brenda Rincon, Diamond Y. Bravo, Elisha Arnold, Alexis Meza, Daisy Camacho-Thompson, Chelsea D. Williams

**Affiliations:** ^1^Department of Psychology, University of California, Riverside, Riverside, CA, United States; ^2^Department of Psychology, California State University, Los Angeles, Los Angeles, CA, United States; ^3^Department of Psychology, Virginia Commonwealth University, Richmond, VA, United States

**Keywords:** medical students, field belonging, grade expectations, community support changes, family support changes

Author, Chelsea D. Williams was omitted as an author in the published article. The correct author list reads:

Brenda Rincon^1^, Diamond Y. Bravo^1*^, Elisha Arnold^1†^, Alexis Meza^1†^, Daisy Camacho-Thompson^2^ and Chelsea D. Williams^3^

^1^Department of Psychology, University of California, Riverside, Riverside, CA, United States

^2^Department of Psychology, California State University, Los Angeles, Los Angeles, CA, United States

^3^Department of Psychology, Virginia Commonwealth University, Richmond, VA, United States

The Author Contributions Statement has been corrected to read:

BR: Conceptualization, Formal analysis, Investigation, Visualization, Writing – original draft, Writing – review & editing. DB: Conceptualization, Data curation, Formal analysis, Funding acquisition, Investigation, Methodology, Supervision, Writing – review & editing. EA: Visualization, Writing – original draft. AM: Writing – original draft. DC-T: Conceptualization, Data curation, Methodology, Writing – review & editing. CW: Conceptualization, Investigation, Writing – review & editing.

The original version of this article has been updated.

